# Quantitative Compositional Analyses of Calcareous Rocks for Lime Industry Using LIBS

**DOI:** 10.3390/molecules27061813

**Published:** 2022-03-10

**Authors:** Juri Agresti, Carlo Indelicato, Matteo Perotti, Roberto Moreschi, Iacopo Osticioli, Ilaria Cacciari, Andrea Azelio Mencaglia, Salvatore Siano

**Affiliations:** 1 Istituto di Fisica Applicata “N. Carrara”, Consiglio Nazionale delle Ricerche, 50019 Florence, Italy; i.osticioli@ifac.cnr.it (I.O.); i.cacciari@ifac.cnr.it (I.C.); a.mencaglia@ifac.cnr.it (A.A.M.); 2 Dipartimento di Scienze Fisiche, della Terra e dell’Ambiente, Università di Siena, 53100 Siena, Italy; carlo.indelicato@unisi.it (C.I.); matteo.perotti@unisi.it (M.P.); 3 Unicalce S.P.A., Via Tonio da Belledo, 30, 23900 Lecco, Italy; rmoreschi@unicalce.it

**Keywords:** LIBS, MLP-ANN, quantitative analysis, lime, calcareous material, calcium oxide, LIBS mapping

## Abstract

Here, the potential of laser-induced breakdown spectroscopy (LIBS) in grading calcareous rocks for the lime industry was investigated. In particular, we developed a system equipped with non-intensified detectors operating in scanning mode, defined a suitable data acquisition protocol, and implemented quantitative data processing using both partial least squares regression (PLS-R) and a multilayer perceptron (MLP) neural network. Tests were carried out on 32 samples collected in various limestone quarries, which were preliminarily analyzed using traditional laboratory X-ray fluorescence (XRF); then, they were divided into two groups for calibration and validation. Particular attention was dedicated to the development of LIBS methodology providing a reliable basis for precise material grading. The congruence of the results achieved demonstrates the capability of the present approach to precisely quantify major and minor geochemical components of calcareous rocks, thus disclosing a concrete application perspective within the lime industry production chain.

## 1. Introduction

Limes represent a wide set of materials used in a growing variety of processes. Three main primary types of limes can be distinguished: (1) quicklime (CaO, the basis of all lime products available on the market), which is achieved by calcining limestone (CaCO_3_-rich sedimentary rocks) and more general calcareous materials in lime kilns around 1000 °C; (2) hydrated or slaked lime Ca(OH)_2_; (3) dolomitic lime (CaO with variable content of MgO), achieved by calcining dolomitic limestone. Various lime products are available on the market, according to the purity grade and type of impurities contained in their corresponding raw materials, final microstructure, and possible mineral and composite mixing. Examples of such products are hydraulic lime, milk of lime, lime putty, and dolime. 

In the last decade, the total yearly production of quicklimes in Europe was around 20 Mt, used in the iron and steel industry (about 40%), constructions and civil engineering (19%), environmental protection (15%), chemical industry (7%), pulp and paper production (6%), agriculture (2%), and others [1,2]. Various limes with optimal compositions are produced for each of these fields of application. In particular, moderate purity grades and suitably balanced contents of CaO and MgO are exploited in raising the pH and supplying the correct content of magnesium to agricultural soils. Similarly, the addition of lime to soils to improve their chemical–physical properties and then to use them for construction requires accurate compositional specifications [3]. Conversely, high grades are used in basic oxygen furnaces to remove impurities (aluminates, phosphorus, silicates, sulfur, etc.) from molten carbon-rich pig iron to transform it into steel, while extra-low-carbon limes are needed in vacuum oxygen decarburization (VOD) to produce stainless steels [4]. During the former process, the viscosity of the slags and their aggressiveness against the lining of the kiln are controlled using suitable dolimes with optimal MgO/CaO content ratios. Lastly, other impurities can also play a crucial role in some lime products, such as hydraulic lime, whose hardening property in water maximizes when it includes clay contents of 20–22% (around 5% of magnesium oxide), which can naturally be achieved by calcining calcareous marls. 

As mentioned above, the purity grade and the microstructure of lime are determined by the composition of the raw calcareous materials and the production processes (sizing, calcination, hydration, removal of undercooked and overcooked components, and others). Usually, petrographic and geochemical investigations are carried out on calcareous materials excavated in a given quarry in order to characterize and quantify their MgCO_3_/CaCO_3_ ratio and impurity components, and then preliminarily define their possible exploitation for achieving specific final lime products [5,6]. Analytical assessments are also carried out on the latter whenever high purity grades and specific physical properties (particle sizes, density, porosity, and its distribution) are required. 

However, as in other raw material production chains, there is a general awareness in the lime industry about the limitations of the traditional analytical approach, based on sampling, sample preparation, and laboratory investigations, due to the associated low representativity, time consumption, and costs. At the same time, the potential of portable spectroscopic techniques allowing for numerous analyses in situ is gradually attracting the interest of the present sector. In particular, the potential of laser-induced breakdown spectroscopy (LIBS) in addressing the crucial problem of the geochemical characterization could allow classifying the calcareous materials, sorting them according to the possible final lime products that can be achieved, minimizing dumping materials, and eventually optimizing the exploitation and sustainability of the quarry. Furthermore, the technique could also be used in quality controls of the final lime products themselves.

The advantages of LIBS with respect to other elemental analysis techniques are well known, in addition to the gradual growth of its exploitation in several sectors (see, for example, [7] and references therein). In particular, LIBS tests on multiphase geological samples have been carried out since the early 1990s [8,9]. Subsequently, the technique was evaluated for the analysis of Mg and Si in Fe ores [10] and sulfide mineral identification in drill core samples [11]. In the last two decades, the rapid discrimination between different species of minerals such as carbonates and silicates (for example, garnets) using correlation [12] and statistical signal processing [13], as well as semi-quantitative drill core scans [14] and rapid quantitative ore analysis and grading [15,16], online sorting, and slurry monitoring, was explored. Furthermore, methods have been implemented for automated quantitative analysis of massive minerals and ores, online analysis of sulfur in Cu and Ni ores, gypsum, anhydrite, and barite [17,18], monitoring of small variations in Si, Ca, Mg, Al, and graphitic C contents in iron ore slurries [19], and online analysis of coal ash. These and other applications were summarized in various review papers ([20,21] and references therein).

To properly address the mentioned sorting and quality control goals, very versatile and low-cost LIBS tools allowing reliable quantitative measurements within the quarry and the lime production area are needed. Handheld LIBS devices currently marketed are mostly suited for metal analyses and do not provide a satisfactory response to such a technological demand. These instruments present limitations in quantitative analyses because they are usually equipped with low-peak-power excitation lasers combined with low-sensitivity and low-resolution spectrometers [22], which provide unsatisfactory performance in possible practical applications within the present industrial sector. Furthermore, commercial handheld LIBS devices do not allow for reliable data acquisition in scanning mode and quantitative elemental mapping, which are needed whenever analyzing raw materials, in order to achieve meaningful average compositions over representative areas. 

In the present work, we exploited our experience in the development and application of portable LIBS devices in the field of cultural heritage [23,24] and mineral exploration [25] in order to approach practical characterization problems of the calcareous materials used in lime industry. A versatile portable LIBS was designed and built, to be used as both a handheld and a tabletop analytical tool, according to different operative setups, and then calibration and validation tests were successfully carried out. In particular, quantitative calibration based on partial least squares (PLS) regression [26] and an MLP neural network [27,28], and final validation were achieved using calcareous samples from lime production quarries provided by the company UNICALCE S.p.A., the main Italian company of lime products. The investigation of the potential of the LIBS approach was supported by traditional geochemical analyses and Raman spectroscopy [29]. The present study represents the optimization phase for finalizing a dedicated LIBS tool and associated measurement protocols, which can represent a suitable technological offer that can contribute to increasing the quality and sustainability of the lime production chain.

## 2. Results and Discussion

**Figure 1:**
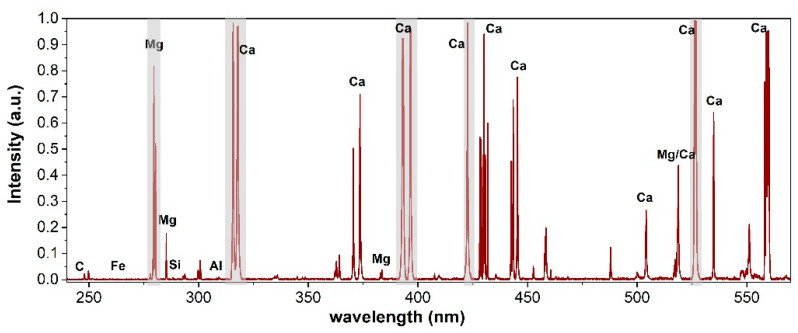
Portion of LIBS spectrum of sample P_31_ with excluded wavelength bands in gray (see text).

A typical LIBS spectrum acquired with the present setup and processed using the SNIP filter is shown in Figure 1. All the main characteristic elements (Ca, Mg, and Si) of limestone are well recognizable, as are the presence of several intense peaks (self-absorbed and prone to saturation), which could severely affect the quantification model if kept among the variables. In particular, their anomalous contribution to the total integral and then to the normalization could seriously affect the robustness of the calibration of the minor elements especially in the linear PLS approach. Thus, as mentioned in Section 3.3.1, they were not included in the PLS data processing. The gray bands in Figure 1 show the spectral intervals which were ruled out: 277–282, 314–320, 391–398, 421–424, and 516–520 nm. 

In order to assess the impact of such spectral selection and normalization on the calibration accuracies, several PLS regression models obtained using the averaged spectra, the averaged band-excluded spectra, the averaged normalized spectra, and the averaged normalized band-excluded spectra were compared. The results are shown in Table 1. 

The RMSE of calibration for the low-concentration oxides SiO_2_, Al_2_O_3_, and Fe_2_O_3_ decreased from the former data treatment to the latter, whereas the minimum RMSE of calibration for the major oxides was obtained for the third data treatment, which was expected since the band exclusion eliminated the most intense peaks of Ca and Mg. Taking into account the overall accuracy of the calibration model, band-exclusion normalization was selected as the data treatment for all oxides.

The PLS regression optimization described in Section 3.3.1 produced the best calibration model reported in Table 2. 

With regard to the ANN, it is useful to provide some further details. In particular, since the hidden neurons can influence the error on the neurons to which their outputs are connected, architectures with different number of hidden layers were explored. The evolution of the error during each epoch (learning curves, LCs) corresponding to the considered architectures was calculated during the training phase with both training and testing subsets (80% and 20% of the input dataset, respectively). We found that increasing the number of hidden layers from one to three showed an improvement in terms of accuracy, at the cost of an increase in the training time. Moreover, the impact on the accuracy of the degree of randomness introduced in the algorithms was also investigated. For each of the mentioned architectures, the training phase with the same hyperparameters was repeated five times by changing the random shuffling and the splitting of the input data. In all the considered topologies, the LCs showed a monotonic decrease without relevant oscillation, and no overfitting or underfitting phenomena were observed. After setting three hidden layers, it was also observed that increasing the number of neurons in the first hidden layer up to 60 did not correspond to a decrease in the prediction accuracy. Thus, the number of neurons of the mentioned layers was set to 50, 45, and 35, and the ANN has been trained as described in Section 3.3.2.

**Figure 2:**
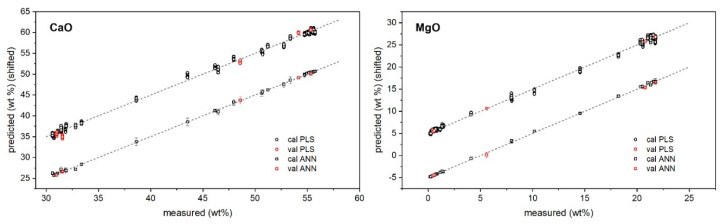
PLS and ANN predicted vs. reference concentrations of major oxides. Plots are vertically shifted for better clarity: ±5 (+PLS, −ANN).

**Figure 3:**
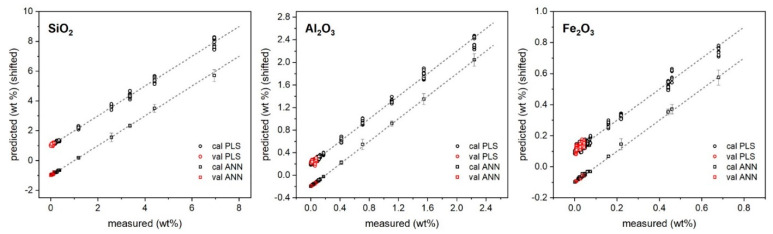
PLS and ANN predicted vs. reference concentrations of minor oxides. Plots are vertically shifted for better clarity: SiO_2_ ± 1, Al_2_O_3_ ± 0.2, Fe_2_O_3_ ± 0.1 (+PLS, −ANN).

The predictions of major and minor oxide contents provided by PLS and ANN regressions versus their measured values are reported in Figure 2 and Figure 3, while the specific results for the validation samples are also listed in Table 3. 

As shown, both approaches returned a satisfactory agreement with reference values provided by XRF. In particular, for major oxides, PLS regression seemed to provide better precision (standard deviation/average below 10% as average) than ANN; for trace oxides, ANN seemed to provide the lower one (below 30% as average). Regarding the accuracy ((predicted − measured)/predicted concentrations), the results seemed to favor ANN regression, which provided the following average relative error of prediction: below 5% for CaO and MgO, and below 20% for the other oxides. This could be expected since the ANN has a higher degree of flexibility to model the intrinsic nonlinearity of the signal induced by self-absorption and the matrix effect [30,31].

The possibility of using LIBS without sample preparation or at least avoiding complex preparation processes significantly extends the potential of the practical exploitation of the technique in lime industry production chain. Thus, after LIBS calibration and validation using pressed powder pellets, the extension of the validation to the analysis of the rock slice samples (S_2–33_) was investigated using the PLS regression.

The main difficulty to be faced when moving from pellets to slices concerns, obviously, the significant inhomogeneity of the latter (rock bulk as it is) with respect to the former (finely ground rock powder, mixed, and pressed). Moreover, for rock slices, the elemental composition as measured within the laser spot could be very different with respect to the calibrated ranges, as determined using powder pellets, which could provide unreliable extrapolation of the model [32,33,34]. These problems were addressed by averaging a large number (N) of LIBS spectra (~1000), which were collected while moving the laser spot within a representative area (around 1 cm^2^, although rather larger areas could be needed in general). The spectra were processed using the same procedure adopted for calibration. In particular, they were grouped by 50 and averaged in order to achieve *n* = *N*/50 average spectra, which contained compositional information from 50 different sites each. The PLS model developed to predict the composition associated with the *n* average spectra was, hence, applied, and the overall average and the standard deviation of the prediction of the whole measurement run were eventually calculated.

**Figure 4:**
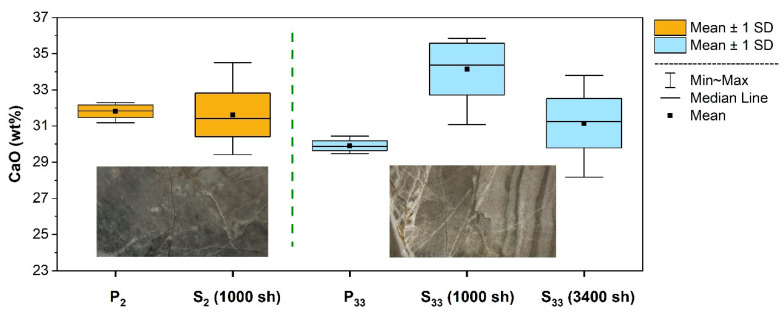
Comparison between LIBS measurements performed on a rather homogeneous rock slice (S_2_) along with the corresponding pellet (P_2_) and on a markedly veined rock slice (S_33_) along with the corresponding pellet (P_33_) using different numbers of laser shots (sh). In each group, the spectra were preprocessed and averaged in the same way (background subtraction, band selection, normalization, averaging 50 measurements). The sizes of the rock slice images are about 25 × 45 mm^2^.

The comparison between powder pellets and corresponding slices (Figure 4) showed that, for a rather homogeneous sample, such as S_2_, 1000 random LIBS analyses were sufficient for the calculated composition to fall within a standard deviation of the composition of the associated powdered sample (P_2_). Conversely, for a rock slice with more pronounced veins, such as S_33_, the same number of analytical shots (1000 sh) did not reproduce its composition within one standard deviation error envelope. In this case, the average composition resulted to be close to the expected value when extending the random scan within the representative area to 3400 sh.

The behavior described above was further investigated in order to determine the number of laser shots needed for representative analytical sampling of an inhomogeneous limestone using the cumulative averaged spectrum for compositional evaluation and monitoring its evolution along a number of random measurements over its surface. 

**Figure 5:**
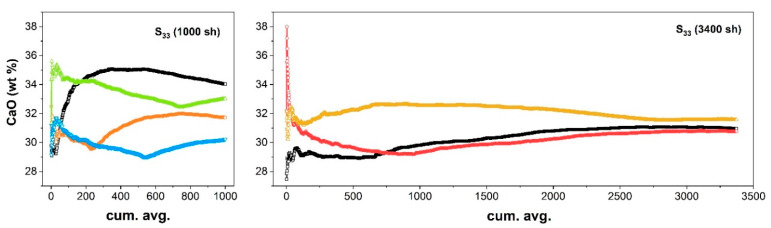
CaO concentration as predicted using the cumulative averaged spectrum in random scans of 1000 sh (left) or 3400 sh (right) in different areas of the nonhomogeneous rock slice (S_33_).

As shown in Figure 5 (left), the CaO content as calculated using the cumulative averaged spectrum ranged between 29–35 wt.% depending on the measurement site and the number of spectra accumulated, and it did not reach a unique stable value. In fact, the slope of the different curves at the extremal part of the graph indicates that the sensitivity to the inclusion of new spectra was still high. On the other hand, increasing the number of measurements above 2500 random sampling (Figure 5, right) produced an estimation of the CaO content close to the expected bulk value, with an almost flat profile, indicating that the averaging procedure reached an almost stable value, which was assumed as representative of the average composition of the area sampled by those measurements.

**Figure 6:**
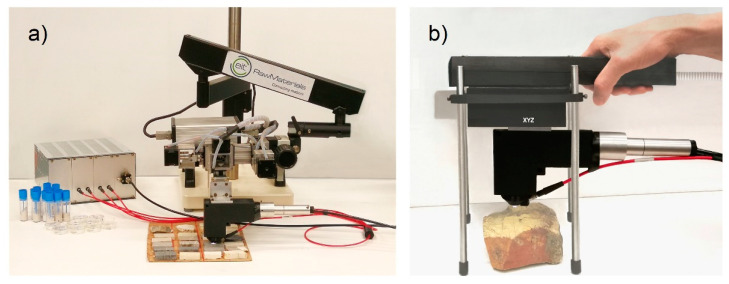
LIBS setups: (**a**) with the endpiece on a XYZ translation group placed on an articulated arm, (**b**) with the endpiece on a handheld XYZ translation group.

**Figure 7:**
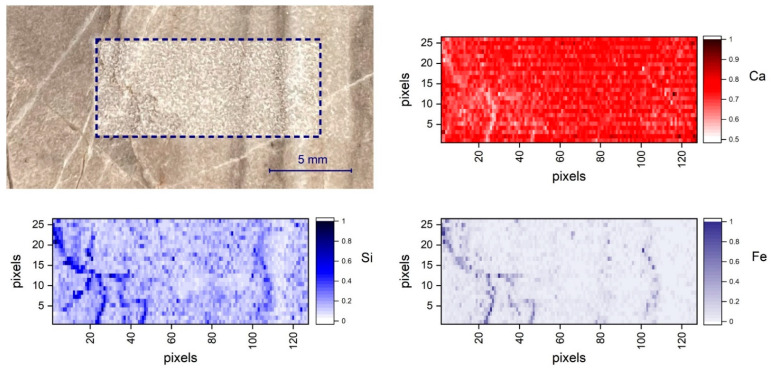
Ca, Si, and Fe LIBS elemental maps corresponding to a scanned area of 15 mm × 5.2 mm of sample S_33_.

It is evident that the number of measurements required to achieve a representative average spectrum which reproduces the composition of the homogenized sample (powder pellet) of the same rock depends on the spatial scale of inhomogeneities, sampling pattern, and the laser spot size [35]. The scanning system of Figure 6a was finally used in order to achieve a standard sampling pattern and averaging principle for local composition assessment. As an example, two-dimensional elemental distribution was measured by scanning the sample S_33_ over a rectangular area of 15 mm × 5.2 mm, as shown in Figure 7. The net intensities of the emission lines of each element (Ca 430.25 nm, Mg 285.29 nm, Si 288.24 nm, Fe 275.01 nm, Al 308.22 nm) were, therefore, used to indicate the corresponding elemental concentration. Range scaling was used to normalize the magnitude of the signal of each element between 0 and 1, since it usually varies considerably from one element to the other, as is the case for the main constituent Ca and Mg, with respect to the trace elements Fe and Si. Figure 7 shows the Ca, Si, and Fe distribution on the 125 × 26 pixel map. 

Quantitative analysis of the scanned area was performed using the cumulative averaged spectra instead of the single-pixel spectrum because the significant compositional variability within the present mineralogic texture could produce unreliable chemical content predictions when using the calibration model described above. To some extent, the cumulative average can be considered more assimilable to the measurement performed on the corresponding powder pellet. 

**Figure 8:**
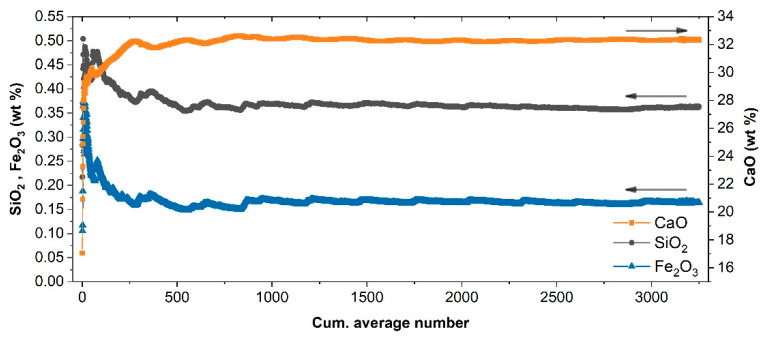
Estimated concentration applying the calibration model to the cumulative averaged spectrum of the 2D scan of Figure 7 on sample S_33_.

Quantitative analyses achieved using the cumulative average of the map’s spectra are shown in Figure 8. The predicted oxide concentrations exhibited large fluctuations in the beginning of the averaging process, mirroring the variability of the element distribution on the sampled surface. After several hundred measurements, the predicted composition stabilized to what can be defined as the local average composition over the sampled area.

It is interesting to observe that, while the CaO estimation was compatible with the bulk value of S_33_, Fe_2_O_3_ and SiO_2_ predictions were higher than the corresponding bulk values. Such a discrepancy should not be considered surprising since the visual inspection of the scanned area confirmed that the sampled surface intercepted macroscopic mineral veins and stylolites with high content of Si and Fe. From the application standpoint, this means that, as for any analytical technique, the reliability of the average values predicted mainly depends on the accuracy of the representative sampling and the homogeneity of the samples themselves.

## 3. Materials and Methods

### 3.1. Setup

According to the versatility needs mentioned above and the examination of the present exploration, excavation, and processing scenarios, we designed and built a basic LIBS system equipped with the following components: (A) a small and very light fiber-coupled endpiece allowing for easy handling, which includes beam delivery optics and mechanical connections for housing the laser excitation and signal collection optical fiber tips; (B) an instrumental module enclosing the laser excitation source and a set of four spectrometers (Avantes B.V., Apeldoorn, The Netherlands) equipped with a Czerny–Turner monochromator and CCD detector array, thus covering the range 200–630 nm with 0.06–0.2 nm spectral resolution; (C) umbilical line connecting the mentioned modules, with one power fiber to transmit the laser beam and a bundle of four fibers (200 μm core diameter) to collect the LIBS signal (at about 45° with respect to the optical axis) and to couple it to the spectrometers. The excitation source used was a SSD QS Nd:YAG (Quantel USA, Bozeman, MT, USA) (1064 nm), maximum 50 mJ/pulse, about 7 ns pulse width, 20 Hz maximum pulse repetition frequency, which was coupled in a 910 μm core diameter optical fiber using a plano-convex lens (120 mm focal length). Within the mentioned endpiece, the laser beam was re-collimated and focused onto the target using two lenses (50 mm and 15 mm focal length, respectively). In this way, the pulse energy coupled to the target was about 20 mJ/pulse, while the laser spot diameter was about 300 μm (intensity on target about 4 GW/cm^2^). The spectrometer acquisition was activated by a homemade optical-trigger board driven by the laser emission, and the integration time was set to 2 ms. 

Due to the natural compositional variability of sedimentary rocks and ores, a number of LIBS point measurements need to be collected and averaged in order to achieve meaningful values of the average local elemental content over a given area. Although, in some cases of homogeneous limestone, a few measurements could be enough to get sufficiently stable average values, in general, at least one complete LIBS scan of the area of interest should be executed [36], after removing powder and any other incoherent deposit from the latter. In cases of pronounced inhomogeneity, several scans of the same area (3D LIBS, [37,38,39,40]) could significantly improve the quantitative geochemical information. For these reasons, the above-described instrument was adapted as a tabletop transportable scanning system by mounting the mentioned endpiece on an XYZ translation group placed on an articulated arm, as shown in Figure 6a, thus achieving a setup that can be easily exploited in the compositional characterization of core samples and rock fragments of any shape. The XYZ translation group allowed micrometric resolution positioning with a scanning range of 50 mm along the X- and Y-axes and 25 mm along the *Z*-axis. Operating the system at 20 Hz allowed obtaining a 10 × 10 mm^2^ LIBS map with a pixel size of 120 × 200 μm^2^ in 230 s (4200 spectra). A homemade software (in LabView^®^) was developed for synchronizing the laser, motors, and spectrometer and for analyzing the LIBS data for elemental maps production. 

Such a system, which was used to investigate the present calcareous rock samples, can easily be adapted in a portable tool suitable for in field measurements thanks to the light weight of the instrumental module (about 3 kg) and of the endpiece (about 300–400 g). However, according to the above-reported considerations, the scanning mode in geochemical studies is not just an option but rather a strict need. Thus, after defining the methodological approach with the present study, we foresee the finalization of a field LIBS also including compacted scanning stages, as shown in the figurative simulation of Figure 6b. This will allow using such a tool in areal and in-depth mapping and averaging, which significantly extends the in situ analytical potential of the present technique on a large variety of rocks, as well as buildings, soils, archaeological remains, and others, without any need to smooth asperities and roughness, since XYZ scanning and laser ablation allow the rapid optimization of the LIBS measurements.

### 3.2. Samples

Here, 32 calcareous rock fragments from different limestone quarries were selected. A slice of about 25 × 45 × 25 mm^3^ was cut from each fragment and polished (S_2–33_), then a further adjacent slice was cut from the same fragment and ground into fine powder, which was compacted in a pellet (P_2–33_) with a diameter of 40 mm and thickness of 10 mm using a laboratory press (about 20 ton/cm^2^) and PVA. Moreover, a portion of the powder from each sample was used to produce fused glass beads to be analyzed by XRF. Furthermore, one (P_1_) GFS Chemicals^®^ standard (GFS-400) was included among the reference samples for calibration purposes.

### 3.3. Analytical Methods

All the glass beads were formerly analyzed using wavelength-dispersive X-ray fluorescence (WD-XRF, benchtop Rigaku Supermini200 spectrometer (Rigaku Europe SE, Neu-Isenburg, Germany) operating at 50 kV and 4 mA); then, LIBS measurements were carried out on the respective pressed pellets using the setup of Figure 6a. The XRF data of 27 glass beads samples, along with the analytical data of the mentioned standard (P_1_), were used for calibrating the LIBS measurements, while the remaining five pellets were used for validating the whole analytical approach.

LIBS random scans over areas of about 1 cm^2^ using 500 laser shots were carried out on the pellet surfaces in order to investigate their degree of homogeneity and eventually extract representative average compositions. The results achieved were compared with those of the corresponding surfaces of the rock slices using a similar scan method. The XRF geochemical data of the whole set of present samples are summarized in Table 4.

Two independent calibration procedures based on PLS and ANN, respectively, were carried out. PLS is a linear optimization technique often used in quantitative LIBS applications [41,42], where a very large number of input variables (intensity at multiple wavelengths) are used to predict a limited number of output values (elemental concentrations) by a regression model built from a set of calibrated samples. Here, the PLS modeling was optimized through a dedicated pretreatment and normalization procedure.

Among the variety of ANN approaches, the multilayer perceptron regression (MLP-R) architecture was considered since it has been proven to be a suitable technique for a wide range of LIBS data processing problems (see, for example, [43] and references therein). The training phase included two steps: the input data were fed forward through the network; in order to measure how far the resulting output was from the desired one, an error function was then calculated, and propagated back to the previous layers while changing the corresponding weights. The training was repeated a number of times (epochs) set a priori.

#### 3.3.1. PLS Regression Method

Each spectrum collected was subjected to the following processing steps. Firstly, automatic background subtraction using a filter based on a statistics-sensitive nonlinear iterative peak-clipping algorithm (SNIP) [44] was executed. Afterward, regions including possible line saturations or severely self-absorbed peaks were excluded. The spectrum was, hence, normalized to the total intensity, i.e., each wavelength bin was divided by the value of area of the spectrum over the selected spectral ranges. As is well known [45], in general, the normalization to the total intensity reduces the effects of shot-to-shot variations and differences in laser–target energy coupling. On the other hand, it should be underlined that the removal of nonlinear spectral regions before normalization and following PLS processing could have a crucial importance, although this aspect is sometimes neglected in the literature.

Following such a pretreatment, the spectra collected for each calibration sample were split into 10 groups of 50 spectra and then averaged, thus achieving 10 representative spectra per calibration sample.

Five PLS calibration models were generated with the sample P_1–28_ for the major element oxides: CaO, MgO, SiO_2_, Al_2_O_3_, Fe_2_O_3_. The models for the latter three oxides were built using a log-linear transformation of the data, by performing the logarithm of the shifted concentration (log(C+1)), before the PLS regression. Log transformation produces a more accurate calibration for low concentrations, helping to remove any skewness and reducing the impact of extreme high values in the regression procedure.

The performance of the model was evaluated by calculating the standard root-mean-squared error (RMSE) resulting from the predicted concentration with respect to the measured values. This indicator is expected to be as low as possible for a reliable model, and it was used to select the best number of PLS components, as described below. 

For a certain number of components, we performed PLS regression with 10-fold cross-validation on the calibration set followed by a prediction test on the validation set, in order to reduce possible bias and overfitting of the data. The whole dataset of 280 average spectra of calibration was divided into 10 subsets, and PLS regression was iterated in such a way that, in every iteration, we took one subset for testing and the remaining subsets for training (internal cross-validation). Lastly, the root-mean-squared error in cross-validation (RMSECV) was the average value of 10 values of the RMSE calculated during the internal cross-validation process. Each calibration model was then employed to predict the concentration values of the set of known samples different from the calibration set. Then, the root-mean-squared error of prediction (RMSEP) was calculated over the validation set. Increasing the number of components produced a decrease in RMSECV and RMSEP until a minimum RMSEP was reached, which set the optimum number of PLS components [46]. We briefly investigated the accuracy of LIBS calibration with or without normalization and found that the abovementioned procedure reduced the mean squared error (MSE) of calibration of CaO by about a factor of three.

#### 3.3.2. MLP Regression Method

Before feeding the network, the data were pretreated. In order to reduce the complexity of the problem, the large number of available spectra for each sample was reduced from 500 to 25 by performing an average operation. This allowed managing 700 spectra in total. From this dataset, the amplitudes of 13 peaks associated with five elements were considered (Table 5). Thus, the input of the network for training was a 13 × 700 matrix, while its output was a 5 × 700 matrix, where 5 denotes the element concentrations of the calibration samples (P_1_–P_28_ in Table 4).

Since the input and output data did not lie in the same range, the performance of the network was improved with some preliminary operations. The input dataset was standardized to zero mean and a standard deviation of one (standard score), and the output dataset was scaled in order to match the scale of the activation function considered. In this case, since the activation function was chosen as a sigmoid, the output dataset was normalized to fall in the range 0–1. Moreover, the maximum number of hidden layers was set to 3, and the learning parameters (learning rate and momentum) were both set to 0.9 and kept constant during training. The root-mean-squared percentage error was considered as the error function.

Before starting the training, the input dataset was randomly shuffled and randomly split into two subsets for training and testing, with different proportions (80% and 20%, respectively). Only the training subset was used for weight updating. During the training phase, the “quality of learning” was evaluated on both training and testing datasets (the latter is not part of the training phase i.e., weight updating), calculating the corresponding learning curves [47,48].

After the network was completely trained (updating hyperparameters using only training dataset), a new dataset was used in order to provide the gold standard to evaluate the final trained model. This allowed giving a measure of how well the model was generalizing, i.e., how accurate the model would be when presented previously unseen data to the trained network. This unseen dataset was obtained from LIBS measurements performed on the five validation samples shown in Table 4. Again, the same pretreatment conducted for the input dataset was also performed on the validation data, resulting in 25 spectra for each validation sample. The input data were, hence, represented by a 13 × 125 matrix (no. of peaks × no. of spectra of the test samples), and the output data were represented by a 5 × 125 matrix.

## 4. Conclusions

Here, the possibility to exploit LIBS as a geochemical tool in order to grade limestone and magnesian limestone, to quantify their impurities, and to perform other quality controls along the lime production chain was experimentally demonstrated on a representative set of calcareous rocks collected in lime quarries. The work proposed a simple and versatile LIBS setup and showed its potential in providing local compositional data over a given area or set of areas of a given sample through calibration, scanning, and averaging of suitable numbers of spectra. Both statistical (PLS regression) and artificial neural network (MPL regression) approaches were proven effective models for elemental calibration using a set of reference samples compositionally similar to those to be investigated. At the same time, the present study evidenced and underlined the need to collect thousands of LIBS spectra and to feed the mentioned models using suitable averages allowing for maintaining the quantifications within the calibration ranges. 

Furthermore, this analytical approach along with the punctual nature of the technique (spot sizes of ~10–100 μm) also allows significantly increasing its analytical sensitivity whenever investigating microgranular mixtures and/or samples including strong concentration gradients on the crystalline texture scale, such as those of many rocks. In this case, limits of detection (LODs) of a few ppm can easily be achieved using a scanning LIBS system equipped with non-intensified sensors, like the one used in this work. Conversely, in general, it is practically impossible to build calibration regressions covering the actual content fluctuations of trace elements, in which case the preliminary average of a number of spectra before feeding the model represents a crucial step. Lastly, the results presented show that the best analytical protocol was achieved through the following operative steps: acquisition in random or mapping scanning mode, sequential cumulative average, corresponding sequential model prediction, and plotting. This should be iterated over a representative area or a set of areas, preferably flat and cleaned, for a sufficient number of laser shots in order to observe the stabilization of the various elemental contents. In this way, LIBS compositional analyses can become significantly competitive with respect to laboratory XRF and ICP, in terms of both costs and analytical capabilities.

Considering the results of the present work, we are now developing a portable and low-cost LIBS device dedicated to geochemical analysis in the lime production industry.

## Figures and Tables

**Table 1:** Relative variation of RMSECV in PLS models when using different data treatments with respect to averaging spectra only. Positive and negative values represent an increase and decrease in the RMSECV, respectively.

Oxide	Bands Exclusion + Avg.	Norm. + Avg.	Bands Exclusion + Norm. + Avg.	PLS Factors
CaO	+29%	−37%	−20%	7
MgO	+44%	−64%	−43%	8
SiO_2_	−22%	−36%	−57%	18
Al_2_O_3_	−21%	−55%	−73%	17
Fe_2_O_3_	−22%	−46%	−56%	13

**Table 2:** PLS model summary.

Oxide	Calibration Range (28 Samples) (wt.%)	Validation Range (5 Samples) (wt.%)	RMSECV (wt.%)	RMSEP (wt.%)	PLS Factors
CaO	30.61–55.74	31.01–55.34	0.57	0.91	7
MgO	0.26–21.79	0.43–21.77	0.44	0.82	8
SiO_2_	0–6.95	0.003–0.14	0.085	0.057	18
Al_2_O_3_	0–2.24	0.009–0.076	0.033	0.038	17
Fe_2_O_3_	0.002–0.68	0.007–0.045	0.019	0.015	13

**Table 3:** Oxide concentration as determined using PLS and ANN calibration. Average and standard deviation (between brackets) are reported for each validation sample.

	CaO		MgO		SiO_2_		Fe_2_O_3_		Al_2_O_3_	
	PLS	ANN	PLS	ANN	PLS	ANN	PLS	ANN	PLS	ANN
P_29_	30.72 (0.27)	31.05 (0.48)	21.63 (0.37)	21.64 (0.17)	0.17 (0.03)	0.10 (0.01)	0.047 (0.016)	0.040 (0.008)	0.061 (0.021)	0.070 (0.007)
P_30_	47.89 (0.24)	48.96 (0.82)	6.86 (0.22)	5.25 (0.86)	0.024 (0.029)	0.084 (0.012)	0.052 (0.012)	0.047 (0.009)	0.01 (0.01)	0.052 (0.034)
P_31_	55.65 (0.09)	55.36 (0.32)	0.31 (0.12)	0.42 (0.25)	0.005 (0.021)	0.004 (0.002)	0.006 (0.007)	0.006 (0.001)	0.018 (0.006)	0.012 (0.002)
P_32_	54.89 (0.10)	54.37 (0.15)	0.85 (0.11)	0.61 (0.24)	0.10 (0.01)	0.030 (0.018)	0.019 (0.011)	0.008 (0.001)	0.058 (0.015)	0.023 (0.012)
P_33_	29.91 (0.27)	31.83 (0.40)	21.97 (0.30)	20.44 (0.34)	0.14 (0.02)	0.177 (0.037)	0.033 (0.014)	0.022 (0.005)	0.081 (0.024)	0.071 (0.007)

**Table 4:** Constituent oxides (wt.%) of samples under investigation and of the standard P_1_, as measured using WD-XRF.

**Calibration** **Samples**	**CaO**	**MgO**	**SiO_2_**	**Fe_2_O_3_**	**Al_2_O_3_**
P_1_	30.610	21.570	0.070	0.050	0.030
P_2_	31.481	21.744	0.059	0.037	0.053
P_3_	31.100	21.787	0.000	0.018	0.009
P_4_	46.170	8.010	0.040	0.030	0.040
P_5_	50.640	1.150	4.410	0.460	1.550
P_6_	30.750	21.380	0.050	0.050	0.040
P_7_	47.960	1.470	6.950	0.680	2.240
P_8_	52.760	0.860	2.580	0.220	0.710
P_9_	54.930	0.580	0.290	0.020	0.130
P_10_	31.860	20.330	0.060	0.070	0.040
P_11_	43.540	10.170	0.370	0.060	0.070
P_12_	50.730	4.120	0.250	0.030	0.110
P_13_	33.380	18.220	1.180	0.160	0.420
P_14_	30.900	21.047	0.205	0.045	0.127
P_15_	31.430	21.322	0.167	0.037	0.095
P_16_	46.470	7.977	0.148	0.049	0.093
P_17_	38.637	14.548	0.347	0.075	0.176
P_18_	53.380	1.333	0.193	0.048	0.110
P_19_	55.220	0.420	0.119	0.024	0.060
P_20_	55.530	0.301	0.025	0.016	0.022
P_21_	55.600	0.192	0.100	0.032	0.072
P_22_	55.750	0.590	0.082	0.016	0.043
P_23_	31.920	20.575	0.173	0.031	0.111
P_24_	32.820	20.505	0.034	0.007	0.030
P_25_	54.730	0.822	0.193	0.045	0.081
P_26_	51.220	0.717	3.366	0.442	1.112
P_27_	55.130	0.390	0.033	0.009	0.013
P_28_	55.350	0.258	0.004	0.002	0.000
**Validation** **Samples**	**CaO**	**MgO**	**SiO_2_**	**Fe_2_O_3_**	**Al_2_O_3_**
P_29_	31.013	21.766	0.114	0.035	0.076
P_30_	48.574	5.602	0.094	0.045	0.060
P_31_	55.339	0.432	0.003	0.007	0.010
P_32_	54.155	0.580	0.031	0.009	0.019
P_33_	31.568	20.787	0.140	0.027	0.074

**Table 5:** List of emission lines considered for training/testing MLP network.

Wavelength (nm)	Element	Wavelength (nm)	Element
251.61	Si	309.3	Al
259.94	Fe	393.37	Ca
274.91	Fe	396.85	Ca
279.55	Mg	422.67	Ca
285.21	Mg	438.35	Fe
288.16	Si	517.27	Mg
308.21	Al		

## Data Availability

The data that support the findings of this study are available from the corresponding author upon reasonable request.

## Sample Availability

Samples of this study are available from the authors.
